# Cerebral edema after ischemic stroke: Pathophysiology and underlying mechanisms

**DOI:** 10.3389/fnins.2022.988283

**Published:** 2022-08-18

**Authors:** Yuhang Gu, Chen Zhou, Zhe Piao, Honghua Yuan, Huimin Jiang, Huimin Wei, Yifan Zhou, Guangxian Nan, Xunming Ji

**Affiliations:** ^1^Department of Neurology, China-Japan Union Hospital of Jilin University, Changchun, China; ^2^Beijing Institute of Brain Disorders, Beijing Advanced Innovation Center for Big Data-Based Precision Medicine, Capital Medical University, Beijing, China; ^3^Advanced Innovation Center for Big Data-Based Precision Medicine, School of Biological Science and Medical Engineering, Beihang University, Beijing, China; ^4^Department of Neurosurgery, Xuanwu Hospital, Capital Medical University, Beijing, China

**Keywords:** blood-brain barrier, cerebrovascular disease, cerebral edema, ischemic stroke, pathophysiology

## Abstract

Ischemic stroke is associated with increasing morbidity and has become the main cause of death and disability worldwide. Cerebral edema is a serious complication arising from ischemic stroke. It causes an increase in intracranial pressure, rapid deterioration of neurological symptoms, and formation of cerebral hernia, and is an important risk factor for adverse outcomes after stroke. To date, the detailed mechanism of cerebral edema after stroke remains unclear. This limits advances in prevention and treatment strategies as well as drug development. This review discusses the classification and pathological characteristics of cerebral edema, the possible relationship of the development of cerebral edema after ischemic stroke with aquaporin 4, the SUR1-TRPM4 channel, matrix metalloproteinase 9, microRNA, cerebral venous reflux, inflammatory reactions, and cerebral ischemia/reperfusion injury. It also summarizes research on new therapeutic drugs for post-stroke cerebral edema. Thus, this review provides a reference for further studies and for clinical treatment of cerebral edema after ischemic stroke.

## Introduction

Stroke places a heavy burden on society and families due to its high morbidity, associated disability, and mortality. Ischemic stroke accounts for nearly 76% of all stroke cases (Virani et al., 2021; Deng et al., 2022). Malignant brain edema (MBE) is a serious complication of stroke, with a mortality rate as high as 80% (Battey et al., 2014; Nawabi et al., 2019). Even in patients with non-life-threatening stroke, the severity of cerebral edema is a risk factor for poor prognosis. A recent study has shown that a midline shift greater than 3 mm can independently predict outcomes after ischemic stroke (McKeown et al., 2022). Cerebral edema after stroke is an important cause of the malignant progression of stroke, and is related to adverse outcomes. In this review, we discuss the classification and pathological characteristics of cerebral edema, the role of various molecules and underlying mechanisms, and new therapeutic drugs for management of cerebral edema that develops after stroke, to provide a basis for further studies and for clinical treatment of this condition.

## Classification and pathological features of cerebral edema

Recent studies have focused on the scientific understanding of the malignant progression of stroke and on improvement of the long-term prognosis of stroke patients. The pathological mechanisms of cerebral edema after stroke are summarized as follows:

Edema after stroke is divided into three groups according to the molecular pathophysiology: cytotoxic edema, ionic edema, and vasogenic edema (Yao et al., 2020). Cytotoxic edema occurs rapidly after stroke, and is followed by ionic edema, vasogenic edema, and then mixed edema (Simard et al., 2007; Liebeskind et al., 2019). Cytotoxic edema and vasogenic edema are interdependent. The prolongation of cytotoxic edema induces vasogenic edema and vice versa (Jha et al., 2019).

**FIGURE 1:**
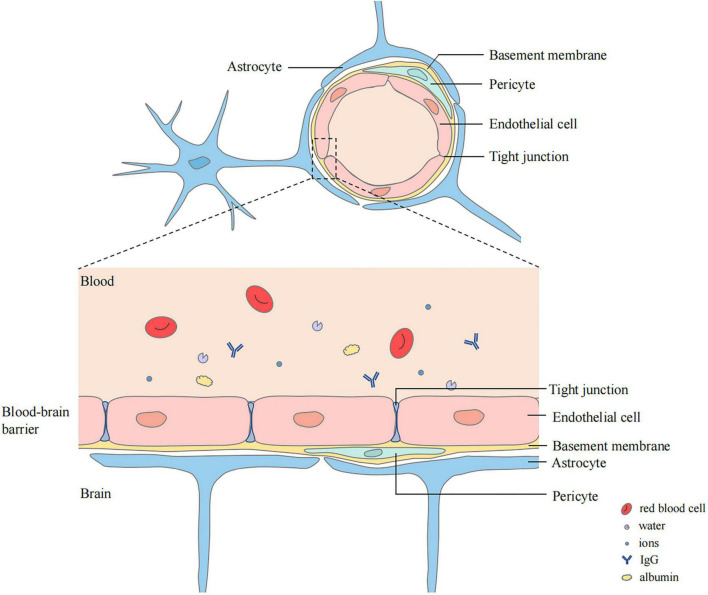
Structure of blood–brain barrier (BBB). BBB locates between the luminal substances of the blood vasculature and the brain interstitium. The capillary lumen is surrounded with endothelial cells connected by tight junctions. Pericytes and Endothelial cells are ensheathed by a basement membrane surrounded by the end-feet of astrocytes.

The blood–brain barrier (BBB) is closely related to cerebral edema. It is a highly selective complex of cells located between the luminal substances of the blood vasculature and the brain interstitium (Stokum et al., 2016; Jiang et al., 2018). It is composed of continuous cerebral capillary endothelial cells, tight junctions between these cells, a complete basement membrane, pericytes, and a glial membrane surrounded by the end-feet of astrocytes (Yu et al., 2020; Ji et al., 2021; Figure 1). It contains transporters that provide nutrients to the central nervous system (CNS), ion transporters that participate in brain ion homeostasis, and efflux transporters that prevent compounds from entering the brain (Jiang et al., 2018). Cerebral ischemia can cause destruction of the BBB. Chemicals, liquids, and blood-borne cells enter the brain parenchyma through the damaged BBB, which destroy the water and ion homeostasis of the brain, resulting in cerebral edema (Keaney and Campbell, 2015).

### Cytotoxic edema

**FIGURE 2:**
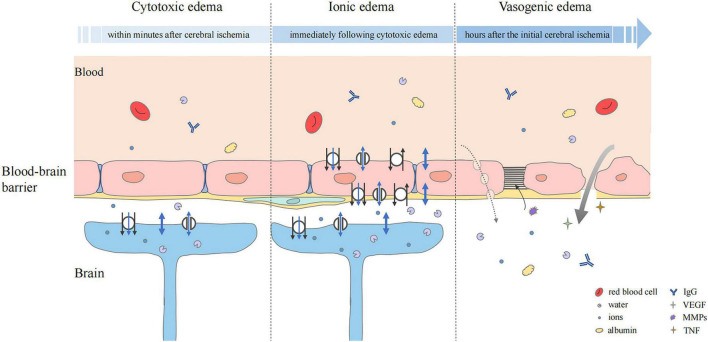
Status of the blood–brain barrier at three phases of cerebral edema. Cytotoxic edema is the initial step and particularly prominent in astrocytes. Cerebral ischemia and hypoxia induced the ion influx (black arrow), which leads to osmotic gradient changes. Water may flow into astrocytes in three ways, simple diffusion (thick blue double-headed arrows), passive transport through transmembrane channels (thin blue double-headed arrows), and water co-transport (blue single-headed arrows). In ionic edema, ion, and water influx are mediated by plasmalemma channels and transporters of endothelial cells. Upregulation of transporters and ion channels also occurs in astrocytes. Vasogenic edema is characterized by destruction of the BBB. The transport of ions, water, and serum proteins such as albumin and IgG may occur directly (thick gray arrow) or *via* pinocytic vesicles (dashed gray arrow). Multiple factors, including VEGF, MMPs, and pro-inflammatory cytokines such as TNF are involved. They mediate neuroinflammation and tight junction degradation, aggravating cerebral edema.

Cytotoxic edema is the initial step in the pathological process of cerebral edema. In the early stage of cerebral ischemia and hypoxia, Na^+^/K^+^-ATPase damage and ion osmotic-gradient changes cause the osmotically active molecules, mainly Na^+^, Cl^–^, and H_2_O, to transfer from outside to inside the cell, leading to cell swelling and providing a driving force for the formation of ionic edema and vasogenic edema (Stokum et al., 2016; Halstead and Geocadin, 2019; Figure 2). This pathological change is particularly prominent in astrocytes (Stokum et al., 2016).

Water may flow into astrocytes in three ways (Stokum et al., 2016). Firstly, simple diffusion *via* the lipid bilayer can lead to the inflow of a large amount of water (MacAulay, 2021). Secondly, water flux is driven by osmotic gradients through transmembrane water channels, including the aquaporin (AQP) family and some astrocyte transporters, such as SGLT1, GLUT1, and GLUT2 (Zeuthen et al., 1997; MacAulay and Zeuthen, 2010). In addition, water can be translocated together with ion fluxes, which is driven by some ion transporters expressed by astrocytes. These transporters, such as NKCC1 and the glutamate transporter EAAT1, can regulate secondary water co-transport through transference of a fixed number of water molecules and ions per transport action (MacAulay and Zeuthen, 2010).

A study in an animal model of ischemia found that extracellular Na^+^ decreased from 141 mmol at baseline to 74 mmol after cerebral ischemia (Mori et al., 2002). When the plasma Na^+^ was 134 mmol, the cytotoxic edema produced a transendothelial Na^+^ concentration differential of about 60 mmol (Mori et al., 2002). The Na^+^ gradient produced by cytotoxic edema acts as a source of potential energy to drive the subsequent inflow of ionic edematous fluid (Stokum et al., 2016).

### Ionic edema

Ionic edema occurs after cytotoxic edema and can develop in the early stage of endothelial dysfunction. Due to the ion concentration gradient formed by cytotoxic edema, Na^+^, Cl^–^, and water are first transported into endothelial cells through the luminal membrane, and then transported outside the lumen through the abluminal membrane of the cerebral capillary endothelial cells (Stokum et al., 2016; Figure 2). Na^+^, the main driver of ionic edema, propels the inflow of secondary participants, such as Cl^–^ and water to balance the electrical and osmotic gradients.

There are three possible ways by which water is transported through the plasmalemma. Firstly, water can move through simple diffusion across the endothelial cell membrane (MacAulay, 2021). Secondly, secondary co-transport of water can be regulated by some common transport channels for water and ions, as well as some endothelial transporters, such as NKCC1, KCC, MCT1, and GAT-1 (Hamann et al., 2010; MacAulay and Zeuthen, 2010). Thirdly, cerebral endothelial cells express some membrane proteins that can regulate passive water transport (Stokum et al., 2016).

### Vasogenic edema

Vasogenic edema occurs after ionic edema and is characterized by destruction of the BBB (Stokum et al., 2016; Chen et al., 2021). With the progress of edema, cross-endothelial permeability pores are formed, through which water and some plasma proteins can be extravasated into the cerebral interstitial compartment (Stokum et al., 2016; Zhang et al., 2020a; Figure 2). Protein and water can enter the interstitial fluid through reverse pinocytosis. Pinocytosis is a biological process in which blood solutes are folded and wrapped by the plasma membrane of endothelial cells to absorb and transport substances (Swanson and Watts, 1995). It is thought that vasogenic edema can also develop through paracellular transport of endothelial cells, which can be triggered by inflammation and cerebral ischemia to increase endothelial permeability (Garcia et al., 1986). There is evidence that the BBB disruption caused by endothelial cell contraction is not an adequate substitute for tight junction disruption (Moy et al., 1996). Endothelial cell contraction may help to enhance the formation of vasogenic edema rather than initiate it. VEGF signaling can generate paracellular permeability pores. When its expression is triggered by cerebral injury, tight junction protein expression is decreased, vascular permeability is increased, and edema formation is enhanced (Kovacs et al., 1996; Skold et al., 2005; Dore-Duffy et al., 2007). A previous study showed that early administration of recombinant VEGF after experimental rat stroke increased edema formation (Zhang et al., 2000).

The development of cerebral edema is a fatal risk factor for adverse outcomes after stroke. Quantifying the severity and evolution of cerebral edema after stroke plays an important but challenging role in clinical studies (Kumar et al., 2022). Midline shift is the most common measurement for cerebral edema and is defined as the maximum deviation of midline brain structures in the axial plane (Ropper, 1986; Vorasayan et al., 2019; Fonseca et al., 2021). It can be assessed by computed tomography (CT), magnetic resonance imaging (MRI), or bedside transcranial sonography (Gerriets et al., 2001; Walberer et al., 2007). However, it is a crude measurement that may be noticeable to substantial cerebral edema and less sensitive to smaller swelling changes. Relative hemispheric volume is defined as 3-dimensional volume ratio of the ischemic hemisphere to the contralateral hemisphere, and seems to be more accurate than midline shift in manifesting the clinical impact of post-stoke cerebral edema (Ostwaldt et al., 2018; Ng et al., 2022a). However, they both measure the mass effect but not water content (Ostwaldt et al., 2018). Net water uptake (NWU) is a CT densitometry-based method used to calculate water uptake in ischemic tissue (Minnerup et al., 2016; Cheng et al., 2022). It symbolizes the proportion of ischemic tissue composed of excess water, which is promising for quantifying the progression of cerebral edema (Broocks et al., 2020). However, the presence of hemorrhagic transformation within cerebral infarction and postangiographic iodine contrast staining might make the measurement of NWU inaccurate, compromising the value of NWU compared to volumetric edema biomarkers (Kumar et al., 2022; Ng et al., 2022b). The entry of water into the cerebral tissues can be visualized using MRI modalities (Obenaus and Jacobs, 2007). T2-weighted imaging (T2WI) and diffusion-weighted imaging (DWI) are two classical sequences used to evaluate edema processes (Obenaus and Badaut, 2022). The apparent diffusion coefficient (ADC), a quantitative parameter of DWI, can be used to evaluate water mobility within the cerebral cortex (Warach et al., 1996). Cytotoxic edema can be observed as early changes in DWI signal intensity. T2WI can visualize increased cerebral water content at later time points during the development of vasogenic edema (Obenaus and Badaut, 2022). However, these MRI techniques are expensive, time-consuming, and cannot monitor cerebral edema in real-time. Electrical impedance tomography (EIT) is a real-time functional imaging technique that allows imaging of electrical impedance changes in the volume of interest (Ke et al., 2022). Because the electrical impedance between cerebral edema tissue and normal cortical tissue is different, EIT could recognize different types of cerebral edema (Yang et al., 2019). However, the application of EIT is limited by factors such as the modeling accuracy and reconstruction algorithms (Ke et al., 2022). The emergence of artificial intelligence points to a new direction for evaluating cerebral edema, but minimal efforts have been made to detect cerebral edema (Obenaus and Badaut, 2022). The application of artificial intelligence in future extensive studies may facilitate the understanding and diagnosis of cerebral edema after stroke.

## Factors associated with the formation of cerebral edema

**FIGURE 3:**
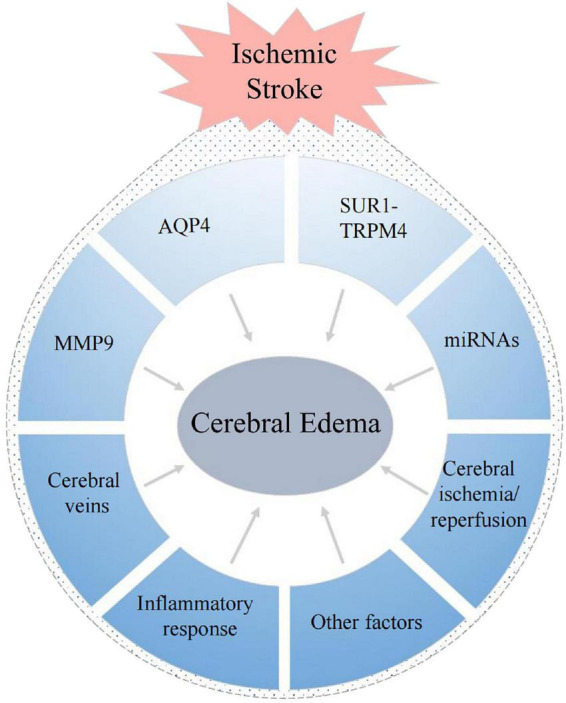
Factors associated with the formation of cerebral edema after ischemic stroke.

The mechanisms of cerebral edema are based on the principle formulated by Starling in the late 19th century (Starling, 1896). He established the basic model of transcapillary driving forces to promote edema formation, and proposed that two elements are needed during the formation of cerebral edema: the driving force, which “pushes” or “pulls” materials into or out of the brain; and the “permeability pores,” which regulate the flux of these materials over the capillary (Simard et al., 2007). There are many factors that can affect these process, and there are often intricate interactions among them (Figure 3). Therefore, the mechanisms underlying cerebral edema are very complex.

Below, we briefly summarize the factors that have been a focus of research in this context.

### AQP4 and cerebral edema

Aquaporin, membrane proteins that allow bidirectional movement of water molecules across the phospholipid bilayer plasma membrane, contain 14 different members, although only AQP1, AQP4, AQP9, and AQP11 are expressed in the CNS (Gorelick et al., 2006; Vella et al., 2015; Stokum et al., 2018). AQP4, which is expressed by astrocytes, plays a bidirectional role in water transport and participates in the formation and elimination of cerebral edema (Stokum et al., 2015). The expression of AQP4 is mainly located in the following four positions in the cerebrum: the end-feet of the paravascular astrocytes, astrocyte processes under the glia limitans external membrane, the ependymal basolateral plasma membrane, and the astrocyte processes under the glia limitans external ependymal membrane (Papadopoulos and Verkman, 2013; Ji et al., 2021).

The end-feet of astrocytes and AQP4 play a vital role in clearance and regulation of cerebral edema (Stokum et al., 2016). AQP4 can also promote the flow of the glymphatic system to eliminate toxic substance deposition in the brain and regulate formation of cerebral edema (Liu et al., 2020). The significantly high expression of AQP4 after ischemic stroke may promote the formation of cerebral edema (Yu et al., 2015; Kitchen et al., 2020; Mestre et al., 2020; Ji et al., 2021). When AQP4 is knocked-out or inhibited, formation of cerebral edema after ischemic stroke is reduced. A study has shown that thyroid hormone therapy can weaken brain edema by inhibiting AQP4 and may have neuroprotective effects on post-stroke patients (Sadana et al., 2015). It has been reported that rat cerebral edema can be reduced by inhaling hydrogen sulfide, involving AQP4 inhibition (Wei et al., 2015). Cerebral edema was found to be alleviated by a combination therapy of AQP inhibitors and cerebrolysin in a permanent middle cerebral artery occlusion (MCAO) animal model (Catalin et al., 2018). After treatment with TGN-020, an AQP4 inhibitor, cerebral edema was reduced at 3 and 7 days in a rat MCAO model after ischemia (Pirici et al., 2017). AQP4 plays a complex dual role during the cerebral edema process after stroke, aggravating cerebral edema formation in the early stage and reducing edema in the later stage (Loh et al., 2019; Clement et al., 2020). However, the mechanisms of this function remain unclear. The use of AQP4 inhibitors after ischemic stroke has become a research hotspot. As the time limit for the formation of cytotoxic edema, ionic edema, and vasogenic edema remains unclear, the timing of initiation and termination of APQ4 inhibitor treatment remains an urgent unanswered question.

### Sulfonylurea receptor 1-transient receptor potential melastatin 4 and cerebral edema

Sulfonylurea receptor 1 (SUR1) belongs to the adenosine 5′-triphosphate (ATP)-binding cassette superfamily encoded by ABCC8 and is a critical mediator of cell swelling in the CNS through the transient receptor potential melastatin 4 (TRPM4) channel (Aittoniemi et al., 2009; Jha et al., 2021; Alquisiras-Burgos et al., 2022). SUR1-TRPM4 is not expressed normally in the CNS, but expressed only after a CNS injury (Stokum et al., 2016; Gerzanich et al., 2019; Alquisiras-Burgos et al., 2020). In all central neurons, CNS injury triggers the activation of the hypoxia-inducible factor 1 transcription factor, which induces the binding of SUR1 to TRPM 4, increases its Ca^2 +^ sensitivity, and makes the channel sensitive to ATP depletion (Woo et al., 2012, 2013; Mehta et al., 2013). The SUR1-TRPM4 channel contributes to the formation of ionic edema by regulating the Na^+^ inflow over the luminal membrane and the Na^+^ outflow over the abluminal membrane. In the case of severe CNS injury and ATP depletion, maladaptive cell swelling and cytotoxic edema can be caused by excessive Na^+^ inflow *via* the SUR1-TRPM4 channel (Stokum et al., 2016). Since the SUR1 regulatory mechanism depends on gene transcription and ATP, it plays a significant role in cerebral ischemia–reperfusion (Stokum et al., 2016). It has been proposed that SUR1-TRPM4 and AQP4 can form a novel heteromultimeric water/ion channel complex, which synergistically regulates rapid and high-volume water flow and astrocyte swelling (Stokum et al., 2018; Jha et al., 2021).

A large number of studies have shown that SUR1-TRPM4 expression is upregulated in ischemic stroke. In a rat model, TRPM4 was upregulated in cerebral endothelial cells 2 h after stroke reperfusion, and inhibition of TRPM4 by siRNA therapy reduced infarct volume and cerebral edema (Chen et al., 2019b). In addition, administration of a TRPM4-specific antibody could prevent the swelling of cells exposed to hypoxia, by inhibiting the channel current (Chen et al., 2019a). In view of the specific driver role of the SUR1-TRPM4 channel in cytotoxicity and ionic edema, pharmacological research on SUR1-TRPM4 has become a hotspot in stroke therapeutic research. Animal experimental studies have shown that glimepiride, a SUR1-TRPM4 channel inhibitor, can reduce stroke in mice and is as effective as glibenclamide in reducing cerebral edema in wild-type mice (Wang et al., 2020c). Currently, a SUR1-TRPM4 channel inhibitor is the only drug that has entered clinical trials for the treatment of cerebral edema after ischemic stroke (Yao et al., 2020). The results of phase II clinical trials have shown that intravenous glibenclamide can reduce cerebral edema and midline displacement (Sheth et al., 2016; Pergakis et al., 2019). Subsequent exploratory studies have shown that glibenclamide can alleviate water accumulation, abate the mass effect, improve the survival rate, reduce midline deviation, and weaken matrix metalloproteinases 9 (MMP9) expressions (Kimberly et al., 2018a; Sheth et al., 2018; Vorasayan et al., 2019). In a clinical study evaluating the efficacy of oral glibenclamide for the treatment of cerebral edema after ischemic stroke, the drug did not increase early death or hypoglycemia, and could prevent cerebral edema (Huang et al., 2019). These findings facilitated the large scale of the phase III clinical trials in large-scale cerebral infarction.

### MMP9 and cerebral edema

Matrix metalloproteinases (MMPs) are a group of zinc endopeptidases that can degrade almost all types of extracellular membrane proteins (Kurzepa et al., 2014). There are over 23 different types of MMPs in the human body. According to their substrate specificity, they are divided into collagenase, gelatinase, matrix metalloelastase, enamel proteinase, and so on (Klein and Bischoff, 2011; Turner and Sharp, 2016). MMPs can mediate the destruction of basement membrane proteins, leading to increased permeability of the BBB, exudation of leukocytes, cerebral edema, and hemorrhagic transformation (Sifat et al., 2017). MMP expression levels are very low under normal conditions, but the levels of MMP2 and MMP9 increase significantly within hours of cerebral ischemia (Zhang et al., 2020d).

A meta-analysis showed that MMP9 levels were higher in patients who suffered a stroke with severe cerebral edema and hemorrhagic transformation (Wang et al., 2020b). The expression of MMPs, and particularly that of MMP9, increases after ischemia. This is strongly associated with extracellular matrix disruption and subsequent vascular permeability (Turner and Sharp, 2016; Beker et al., 2022). MMP9 increases rapidly after cerebral ischemia and hypoxia, destroying the integrity of the vascular wall by degrading tight junctions and the extracellular matrix to increase the BBB permeability, and further leading to neuronal death, cerebral edema, and hemorrhagic transformation (Yang and Rosenberg, 2011; Shi et al., 2016; Bernardo-Castro et al., 2020). Some studies found that MMP9 is involved in maintaining the structural integrity of the AQP4 water channel and participates in the regulation of cerebral edema (Wang et al., 2014; Datta et al., 2022). An *in vitro* study showed that MMP9 silencing downregulated the expression of AQP4 in astrocytes (Li et al., 2020). It has also been suggested that MMP9 is related to the inflammation induced by ischemic stroke and participates in the destruction of the BBB (Bellut et al., 2021; Kim et al., 2021). MMP inhibitors can reduce cerebral edema associated with ischemia by partially preventing the degradation of tight junction proteins (Turner and Sharp, 2016; Datta et al., 2022). Another study has shown that downregulation of MMP9 can reduce destruction of the BBB and cerebral edema in a murine middle cerebral artery occlusion–reperfusion model (Xiong et al., 2022). Some studies have reported that MMP9 may be one of the most promising biomarkers for assessing the BBB permeability and predicting hemorrhage transformation in ischemic stroke (Bernardo-Castro et al., 2020; Mechtouff et al., 2020). However, further research is needed in this regard.

### MicroRNAs and cerebral edema

MicroRNAs (miRNAs), a class of non-coding single-stranded RNA molecules approximately 22 nucleotides in length, encoded by endogenous genes, can regulate gene expression at the transcriptional level and have become promising targets for disease treatments (Carleton et al., 2007; Li et al., 2018). The expression of MiR-1 has been shown to be related to ischemic injury and apoptosis (Chen et al., 2006). The volume of cerebral infarction can be decreased after anti-MiR-1 treatment (Selvamani et al., 2012). The application of a MiR-1 antagonist significantly reduced cerebral edema and BBB damage (Talebi et al., 2019). Notably, studies have shown that other miRNAs, such as miRNA-132 and miRNA-1906, are potential targets for the treatment of cerebral edema (Zuo et al., 2019; Yu and Li, 2020). However, the mechanisms remain unclear and further research is required.

### Cerebral veins and cerebral edema

The intracranial venous system comprises 70–80% of the intracranial circulating blood volume and is the main blood storage and reflux system (Pang, 2001). In recent years, studies of intracranial veins have attracted increasing attention. An increasing number of studies have shown that, during acute ischemic stroke (AIS), the functional reflux of the cerebral vein may be as important as the arterial flow, and that the status of cerebral venous reflux may provide additional information about patients, such as prognosis prediction.

A clinical study showed that hypoplasia or occlusion of the transverse sinus is related to prolongation of intracranial circulation time and impairment of brain autonomous regulation and is positively associated with severe cerebral edema after massive middle cerebral artery infarction (Yu et al., 2009). A clinical study showed that, regardless of the status of the collateral vessels on CT angiography (CTA) after treatment of AIS, the venous outflow was associated with cerebral edema formation. A good venous outflow was related to a reduction of the NWU and good functional outcomes (Faizy et al., 2021b). The team also showed that good venous outflow before treatment was associated with successful reperfusion, favorable tissue-level collaterals, and a good prognosis in AIS patients who received intravascular treatment (Faizy et al., 2021a,c). A previous study has shown that the ipsilateral medullary vein can be an important predictor of adverse clinical outcomes after AIS and is related to hypoperfusion (Yu et al., 2016). In patients with acute large artery occlusion, the absence of the ipsilateral cortical vein as a specific imaging marker of cerebral midline shift contributes to the estimation of the baseline core volume of ischemic stroke in the prediction of a midline shift (Zhang et al., 2020c). A midline shift is more likely to develop in patients with dural sinus hypoplasia (Volny et al., 2016). However, to date, most of these have been clinical studies, and the underlying mechanisms remain unclear.

### Inflammatory response and cerebral edema

Recently, the role of inflammatory response in the BBB destruction in ischemic stroke has been increasingly recognized. Cerebral ischemia manifests as decreased inflammation inhibitory signals and increased alert signals from dead or necrotic neurons cells and glial cells, which may activate quiescent brain immune cells (Liesz et al., 2015; He et al., 2019). Cerebral immune cell activation further upregulates pro-inflammatory factor and chemokine expressions, activates MMPs to damage the integrity of the BBB, enlists peripheral immune cells to the damaged site, promotes the development of irreversible injury in the infarct core area, and results in secondary BBB injury (Malone et al., 2019; Mulder et al., 2021; Qiu et al., 2021). Immune cells, including cerebral immune cells (CICs) and peripheral immune cells (PICs) play a vital role.

After ischemic stroke, PICs, including neutrophils, monocytes, and T lymphocytes, lead to microvascular diseases and the secretion of inflammatory molecules, thereby increasing BBB permeability. In the late stage of ischemic stroke, these contribute to BBB repair and angiogenesis (Qiu et al., 2021). Specifically blocking the adhesion of neutrophils to endothelial cells in venules in the mouse MCAO model was found to significantly reduce the volume of cerebral infarction and neurological deficit and improve both short-term and long-term functional outcomes (Dhanesha et al., 2020). Other studies have shown that neutrophils may express anti-inflammatory phenotypes; the N2 polarization of neutrophils contributes to the phagocytosis of neutrophils by microglia/macrophages, resulting in a reduction of cerebral edema and infarct volume (Garcia-Culebras et al., 2019; Hou et al., 2019). Therefore, neutrophils may play a dual role in the evolution of ischemic stroke.

Similarly, CICs, including microglia, astrocytes, and pericytes of the BBB, play a profound immunomodulatory role in ischemic stroke. Microglia and astrocytes are activated within minutes after cerebral ischemia and release some pro-inflammatory factors, such as TNF-α, NF-κB, IL-1β, and IL-6 (Bonaventura et al., 2016; Kim and Cho, 2016). They promote the inflammatory response, destruction of the BBB structure, increase of the BBB permeability, and uncontrolled MBE or symptomatic intracerebral hemorrhage (Kanazawa et al., 2017). In a rat permanent MCAO model, preconditioning with a TNF-α receptor inhibitor had a protective effect against neurological impairment, cerebral infarction, and edema after stroke (Lin et al., 2021). NF-κB, generally considered as the “central link” of inflammation *in vivo*, is an important mediator in the process of ischemia–reperfusion injury. Inhibition of NF-κB expression can reduce the inflammatory cascade and protect the structure and function of the BBB (Wu et al., 2018; Howell and Bidwell, 2020). After cerebral ischemia, pericytes express an inflammatory phenotype (CD11b-positive), upregulate pro-inflammatory cytokine expression, and promote increased BBB permeability (Zhou et al., 2018). It is thought that the phagocytosis of pericytes may contribute to the regression of inflammation and repair of the BBB in ischemic stroke (Qiu et al., 2021). Therefore, pericytes may play a bidirectional role in ischemic stroke, which needs to be confirmed by further studies.

CICs and PICs are interwoven in a subtle and complex network (Qiu et al., 2021). Moreover, the phenotypes of inflammatory response involved by immune cells are different in the initiation, progression, and regression stages of cerebral ischemia (Mulder et al., 2021). Treatments that target only one kind of immune cell may affect another kind of immune cell and even lead to poor outcomes in ischemic stroke. Therefore, more studies are needed to explore the mechanisms of inflammatory response of cerebral edema after ischemic stroke.

### Cerebral ischemia/reperfusion and cerebral edema

The most effective treatment for AIS is to achieve the recanalization and reperfusion of ischemic brain tissue. Intravenous thrombolysis and mechanical embolectomy are two widely recognized treatment strategies (Rabinstein, 2017; Feng et al., 2019). It is true that many patients benefit from the above treatments, but some patients who receive the above treatments have a poor prognosis. Scholars gradually put forward the concept of cerebral ischemia/reperfusion injury. It is caused by a series of pathological cascade reactions triggered by the recovery of oxygenated blood flow into the ischemic brain tissue (Eltzschig and Eckle, 2011; Fisher and Savitz, 2022; Wang et al., 2022). At present, the relationship between reperfusion and cerebral edema is still not clear, as the views on the effect of reperfusion on cerebral edema are different.

In secondary analysis of a multicenter prospective clinical trial of endovascular treatment for patients with AIS, Kimberly et al. (2018b) showed that successful reperfusion was linked to a reduced midline shift and that reperfusion therapy reduced cerebral edema. However, this analysis was performed in patients using a baseline non-contrast CT and CTA, a follow-up CTA, or magnetic resonance angiogram only, and no perfusion imaging was used to assess core infarct volumes. A meta-analysis of seven randomized controlled trials for AIS reperfusion therapy in 2021 showed that reperfusion therapy was associated with maximal midline shift as a measure of space-occupying cerebral edema in patients with a large baseline core infarct volume (> 130 mL) (Ng et al., 2021b). Ng et al. (2021a) performed a *post hoc* analysis of randomized trials of endovascular therapy for patients with anterior circulation strokes and explored the relationships between pretreatment core and mismatch volume, reperfusion, and cerebral edema after stroke. The patients underwent baseline CT perfusion imaging. Cerebral edema was measured in the midline shift on a 24-h follow-up CT or MRI. Most patients had a small to moderate core volume. This study showed that successful reperfusion was associated with a reduced cerebral edema in patients with small to medium cerebral infarction. Ng et al. (2021a) also found that a large core volume and small mismatch volume were associated with increased cerebral edema after reperfusion, indicating that the effect of reperfusion on post-stroke cerebral edema may vary. To assess the role of ischemic lesion volume after reperfusion treatment in cerebral edema, Ng et al. carried out further research to overcome the limitations of imaging technology and midline shift. They used midline shift and relative hemispheric volume to measure the cerebral edema. The patients underwent 24-h follow-up MRI with dynamic susceptibility contrast-enhanced perfusion-weighted imaging (Ng et al., 2022a). Ng et al. (2022a) found that continuous hypoperfusion for 24 h after reperfusion treatment was related to worse cerebral edema, even though some patients successfully achieved reperfusion. Their series of studies showed that the development of cerebral edema in response to reperfusion may depend on the physiological state of brain tissue, while the irretrievable infarcted tissue swells significantly after reperfusion and the salvable tissue does not swell nor has a low risk of swelling after reperfusion.

In animal experiments, the transient MCAO model showed that there was significant cerebral edema after reperfusion, suggesting that reperfusion injury promoted the formation of cerebral edema (Pillai et al., 2009; Winkler et al., 2021). This may be related to the oxidative/nitrosative stress reaction after reperfusion, in which free radicals play an important role (Sun et al., 2018). An animal experiment in 2022 showed that preventive inhibition of free radical production could reduce cerebral edema in reperfused MCAO rats (Xing et al., 2022). Excessive nitric oxide levels after reperfusion can affect activation of the MMP pathway and the distribution of tight junctions, leading to destruction of the BBB and cerebral edema aggravation (Sun et al., 2018). Oxidative stress promotes the release of pro-inflammatory cytokines, leading to a neuroinflammatory response (as described above), directly or indirectly resulting in destruction of the BBB and cerebral edema (L et al., 2016; Jurcau and Simion, 2021). The mechanism is complex and requires further investigation.

It is apparent that there are inconsistencies between the results of clinical studies and animal experiments. Moreover, some conclusions from animal experiments have not been verified in clinical practice. This may be because the transient MCAO model used in animal experiments rapidly forms a large cerebral infarction in the middle cerebral artery distribution area, demonstrating the adverse effects of reperfusion on cerebral edema. However, in clinical studies, the volume of the core infarction in many patients is relatively small, which reflects the different effects of reperfusion on cerebral edema (Ng et al., 2021b). It is crucial to study the relationship between reperfusion and cerebral edema, clarify the influence of reperfusion on cerebral edema, and explore effective treatment.

## Treatment of cerebral edema

At present, osmotic diuretics, particularly mannitol and hypertonic saline, remain the main drugs used clinically to reduce cerebral edema. The main mechanism involves establishment of an intravascular osmotic gradient, resulting in water flowing from the intercellular space to the intravascular space. The main function of this intervention is to reduce intracranial hypertension and alleviate the mass effect, but it may lead to serious complications, such as kidney injury and water and electrolyte disorder (Zhang et al., 2019; Stokum et al., 2020). Decompressive craniectomy is an effective method for the treatment of MBE, which can reduce mortality and improve prognosis, but most surviving patients are left with severe disability (Shah et al., 2019). Moreover, these interventions can only be applied after complete development of destructive edema, and are high-risk and low-efficacy approaches (Torre-Healy et al., 2012; Gopalakrishnan et al., 2018). Treatments targeting multiple pathways involved in the formation of edema in the CNS, with a view to prevention, may be more valuable than those that eliminate edema once it has developed (Jha et al., 2019).

It is currently a research hotspot to select targets and study new drugs to prevent and treat cerebral edema, based on the underlying molecular mechanisms. A large number of studies have been conducted to investigate these targets. These studies included treatment with SUR1-TRPM4 channel inhibitors (Vorasayan et al., 2019; Wang et al., 2020c), AQP4 blockers (Darabi and Mohammadi, 2017; Catalin et al., 2018), MMP9 inhibitors (Cui et al., 2012; Kimberly et al., 2018a), ion channel blockers, such as NKCC1 and NHE (Spasov et al., 2016; Zhang et al., 2020b), VEGF-related drugs (Yang et al., 2018; Wang et al., 2019), miRNAs (Talebi et al., 2019; Zuo et al., 2019), and some other protective agents, such as edaravone, calcitriol, and 3-aminobenzamide (Liu et al., 2019; Sadeghian et al., 2019; Wang et al., 2020a). However, most of these studies involved animal experiments and few involved clinical trials. Glibenclamide, a potent SUR1-TRPM4 channel inhibitor, is known to enter clinical trials for the treatment of post-stroke cerebral edema. GAMES-Pilot (NCT01268683) is a phase II trial that used intravenous glibenclamide in patients with anterior circulation stroke. A post-exploratory analysis of this trial suggested that glibenclamide was related to decreased water diffusivity and MMP-9 levels, indicating vasogenic edema reduction (Sheth et al., 2014). GAMES-RP (NCT01794182) was a phase II trial that used intravenous glibenclamide to verify the efficacy of intravenous glibenclamide compared with placebo in patients with large anterior hemisphere infarctions at risk of MBE. Post-exploratory analyses of this trial showed that intravenous glibenclamide reduced the midline shift, NWU, and MMP9 expression (Kimberly et al., 2018a; Sheth et al., 2018; Vorasayan et al., 2019). CHARM (NCT02864953), a phase III trial to assess the efficacy and safety of glibenclamide in large hemispheric infarctions for cerebral edema, is currently underway. A phase I trial of AER-271, an aquaporin inhibitor, is currently in progress for its eventual use in patients with AIS (NCT03804476). No specific new drugs have been approved for clinical application.

## Conclusion and perspective

Cerebral edema after ischemic stroke has a high morbidity, mortality, and disability, and is increasingly focused on in research. At present, the treatment of cerebral edema is mainly based on clinical experience, and mostly involves symptomatic treatment after cerebral edema has developed. Such treatment cannot curb the occurrence and development of malignant edema from its basis. Therefore, it is essential to clarify the mechanisms underlying cerebral edema development after ischemic stroke, in order to identify patients prone to MBE early, to find effective therapeutic targets, to determine more effective forms of diagnosis and treatment, and to carry out effective prevention and treatment in time.

## Author contributions

YG designed and wrote the manuscript. CZ contributed to the conception and carried out logic examination of the manuscript. ZP and HY participated in the review of medical professional knowledge of the manuscript. HJ, HW, and YZ helped with proofreading and revision. GN and XJ contributed to the conception and critically revised the manuscript. All authors contributed to the article and approved the final version.
